# Behavior Change Techniques in mHealth Apps for the Mental and Physical Health of Employees: Systematic Assessment

**DOI:** 10.2196/mhealth.6363

**Published:** 2018-10-03

**Authors:** Elsbeth de Korte, Noortje Wiezer, Maartje Bakhuys Roozeboom, Peter Vink, Wessel Kraaij

**Affiliations:** ^1^ Netherlands Organization for Applied Scientific Research Leiden Netherlands; ^2^ Faculty of Industrial Design Engineering Delft University of Technology Delft Netherlands; ^3^ Leiden Institute of Advanced Computer Science Faculty of Science Leiden University Leiden Netherlands

**Keywords:** behavior change techniques, mHealth, mental health, physical health, lifestyle, workplace, app, employee, work

## Abstract

**Background:**

Employees remain at risk of developing physical and mental health problems. To improve the lifestyle, health, and productivity many workplace interventions have been developed. However, not all of these interventions are effective. Mobile and wireless technology to support health behavior change (mobile health [mHealth] apps) is a promising, but relatively new domain for the occupational setting. Research on mHealth apps for the mental and physical health of employees is scarce. Interventions are more likely to be useful if they are rooted in health behavior change theory. Evaluating the presence of specific combinations of behavior change techniques (BCTs) in mHealth apps might be used as an indicator of potential quality and effectiveness.

**Objective:**

The aim of this study was to assess whether mHealth apps for the mental and physical health of employees incorporate BCTs and, if so, which BCTs can be identified and which combinations of BCTs are present.

**Methods:**

An assessment was made of apps aiming to reduce the risk of physical and psychosocial work demands and to promote a healthy lifestyle for employees. A systematic search was performed in iTunes and Google Play. Forty-five apps were screened and downloaded. BCTs were identified using a taxonomy applied in similar reviews. The mean and ranges were calculated.

**Results:**

On average, the apps included 7 of the 26 BCTs (range 2-18). Techniques such as “provide feedback on performance,” “provide information about behavior-health link,” and “provide instruction” were used most frequently. Techniques that were used least were “relapse prevention,” “prompt self-talk,” “use follow-up prompts,” and “provide information about others’ approval.” “Stress management,” “prompt identification as a role model,” and “agree on behavioral contract” were not used by any of the apps. The combination “provide information about behavior-health link” with “prompt intention formation” was found in 7/45 (16%) apps. The combination “provide information about behavior-health link” with “provide information on consequences,” and “use follow-up prompts” was found in 2 (4%) apps. These combinations indicated potential effectiveness. The least potentially effective combination “provide feedback on performance” without “provide instruction” was found in 13 (29%) apps.

**Conclusions:**

Apps for the occupational setting might be substantially improved to increase potential since results showed a limited presence of BCTs in general, limited use of potentially successful combinations of BCTs in apps, and use of potentially unsuccessful combinations of BCTs. Increasing knowledge on the effectiveness of BCTs in apps might be used to develop guidelines for app developers and selection criteria for companies and individuals. Also, this might contribute to decreasing the burden of work-related diseases. To achieve this, app developers, health behavior change professionals, experts on physical and mental health, and end-users should collaborate when developing apps for the working context.

## Introduction

Despite increased awareness and growing efforts to develop measures to effectively manage work-related risk factors and promote workers’ healthy behavior, employees are still at risk of developing physical and mental health problems [1,2]. This is caused by physical and psychosocial work demands and unhealthy lifestyle behaviors, such as low physical activity levels and sedentary behavior. This is often provoked by the way current work and working environments are arranged.

The development of new technologies has brought about many changes in the way people work, resulting in a shift away from occupations that require moderate-intensity physical activity to occupations that are composed of sitting [3,4]. Physical inactivity and sedentary behavior (defined as time spent sitting [4]) are associated with deleterious health effects such as cardiovascular diseases, cancer, type 2 diabetes, and obesity [5-8]. Research has shown that employees with low physical activity levels and sedentary behavior are less productive at work (presenteeism), have decreased workability, and take more sick days [9-12].

Furthermore, the number of employees working with computers has increased over the past decades [13]. Research shows a relationship between computer use and the development of musculoskeletal symptoms [13-15]. Static postures and repetitive movements, physical work demands that are associated with computer work, are related to presenteeism, decreased work ability, and sickness absence [16,17].

During the past decade organizations started to organize work flexibly [18]. Employees decide for themselves where, when, and with which (digital) tools they work. This brings advantages such as autonomy, remote collaboration, and increased possibilities for sharing information. However, there are also drawbacks, such as struggling with managing the inflow of information, interruptions and task switching, perceived pressure to respond quickly, decreased perceived social support, and a disturbed work-life balance [18]. High psychosocial work demands are associated with health complaints, sickness absence, decreased workability, and productivity loss [1,3,19-22].

Improved working conditions are needed to create a healthy and productive working population [10,16]. Besides that, the workplace is a fruitful setting for health promotion because of the presence of natural social networks, the possibility of reaching a large population, and the fact that people spend a great deal of their lifetime at work [9,23,24]. For these reasons, much effort has been put into the development and evaluation of interventions in the workplace setting. This includes selective activities to change the individuals’ risks, attitudes, behavior, and awareness as well as comprehensive interventions such as workplace health promotion programs [1,9,25,26]. However, research shows that workplace interventions may be beneficial, but not all these interventions are useful, or their overall effects are small [1,9,24-32].

Research shows that workplace interventions are more effective when they involve evidence-based principles that (1) offer a variety of engagement modalities, (2) start with a needs assessment of participants, (3) offer higher intensity of contacts to keep participants actively involved, (4) are tailored to address participants’ needs, (5) address multiple risk factors, (6) support self-management, (7) use incentives, (8) provide easy access and easy follow-up, (9) use social support, and (10) are grounded in behavior theory [9,24,28,31,33]. Mobile and wireless technology is a growing area in supporting health behavior change and might offer a promising approach as a workplace intervention since it could offer many of these elements [34-37]. Mobile health, also known as mHealth, covers medical and public health practice supported by mobile devices, such as mobile (smart) phones, personal digital assistants and other wireless devices. It also includes lifestyle and well-being apps that may connect to wearable sensors and personal guidance systems [38]. Various features make them good candidates for the delivery of interventions supporting health behavior change. First, as portable devices, they can continuously monitor the users’ behavior using sensors. They offer the opportunity to bring behavioral interventions into important real-life and working contexts where people make decisions about their health and encounter barriers to behavior change. Second, they may provide cheaper, more convenient interventions. Third, the connectedness facilitates the sharing of data with health professionals or peers. Finally, the increasing ability to use sensors to infer context, such as user location, movement, emotion, and social engagement. This has raised the prospect of timely, tailored interventions for specific contexts [39-43]. As a result, these technologies support a participatory role by users, while enhancing their responsibility for their health and performance [38].

mHealth apps are being developed and evaluated to support behavior change of the general population in a variety of domains, such as physical activity [44-48], obesity [49], and stress management [50-52]. Even with the recent proliferation of apps, research evidence regarding their effectiveness is scarce [53]. The vast majority of commercial apps have not been evaluated using scientific methods, and these apps tend not to be grounded explicitly in theories of health behavior [54]. In recent years, mHealth apps have been developed to target the occupational setting [55-57], a context characterized by its specific barriers. Physical working contexts might put additional constraints on the use of mHealth apps, for instance when working in cleanrooms or high-security settings. Likewise, the organizational working context has specific focus points, such as the fit of an app with working schedules, embedding an app within prevention programs, and the role of management in implementation and adoption of an app. However, despite their potential, little research has been published on mHealth apps for employees. Only 1 study was found showing the positive effects of a tailored mHealth intervention on physical activity, snacking behavior, and sleep among airline pilots [58]. Insight is needed to determine whether mHealth apps are a powerful medium for delivering interventions in the workplace setting. Therefore, these apps need to be evaluated on (1) their potential to support healthy work behavior, (2) their consistency with evidence-based practices, and (3) their effectiveness in improving mental and physical health. The aim of this study is to examine the first step: to assess whether mHealth apps for employees use principles and constructs underlying the processes of behavior change to enhance their mental and physical health.

Research on internet interventions (electronic health) and mHealth shows that they are more likely to be useful if they are firmly rooted in health behavior change theory [34,36,40,59]. Understanding which behavior change techniques (BCTs) are implemented can illuminate mechanisms by which using an app might facilitate behavior change as well as the types of persons for whom a given app may work best [60]. Abraham and Michie [61] and Michie et al [62] suggested several BCTs common to many health behavior theories and developed several versions of a taxonomy to identify BCTs in a range of health promotion interventions [61,62]. The taxonomies have been used to identify techniques or combinations of techniques that might enhance effectiveness [36,40].

A large body of research has been published using the taxonomy in traditional health promotion interventions [40], but few have quantified the extent to which specific BCTs are included in apps. To date, studies have evaluated whether apps for physical activity [40,54,60,63] or apps for physical activity and diet [53] incorporate BCTs. The most frequently applied BCTs in traditional health promotion interventions are “goal-setting,” “prompt intention formation,” “provide feedback on performance,” “self-monitoring,” and “review of behavioral goals” [40,61,64]. Studies report inconclusive evidence regarding the number of BCTs that are associated with effectiveness. A systematic review by Webb et al [59] on Web-based interventions reported that interventions that include a larger number of BCTs, using a taxonomy adapted from Hardeman et al [65], are more likely to be effective. In contrast, another meta-analysis by Michie et al [64] using the Abraham and Michie’s taxonomy [61], suggested that the number of included BCTs is not associated with a larger effect. The study showed that interventions were most likely to be effective when “self-monitoring” was used as a technique, or when “self-monitoring” plus an additional self-regulation technique were used [64].

When interventions involve multiple BCTs, the effects might be additive, neutral (ie, cancel each other out), or amplified [66]. Accordingly, the inclusion of specific combinations of BCTs appears to be critical. Dusseldorp et al [66] used meta-analysis to conclude that specific combinations of BCTs increase the chances of achieving a change in health behavior, while other combinations decrease them. Specific combinations were more successful than average, and the strongest effects were found with motivation-enhancing BCTs. Most effective combinations were “provide information about behavior-health link” with “prompt intention formation” and “provide information about behavior-health link” with “provide information on consequences” and “use follow-up prompts.” Least effective were interventions using “provide feedback on performance” without “provide instruction.”

In summary, studies on traditional health promotion interventions show that not only the presence of BCTs, but also specific combinations of BCTs might explain intervention success. Up until now, none of the studies on the inclusion of BCTs in apps for physical activity and diet [36,40,54,60,63] have evaluated the presence of specific combinations of BCTs. Although this has not yet been confirmed in studies on mHealth in general, and specifically for the occupational setting, it can be suggested that certain combinations of BCTs also serve as an indicator for potential effectiveness in mHealth. This study aims to evaluate whether apps for the mental and physical health of employees incorporate BCTs and, if so, which ones can be identified, and which combinations are present.

## Methods

### Overview

A comparative assessment was made of apps aimed at reducing physical and psychosocial risks at work including stress prevention or coping with stress and to promote a healthy workstyle (ie, prevention of sedentary behavior or promotion of physical activity) for individual workers. Three independent reviewers undertook the assessment of the presence of BCTs and combinations of BCTs in apps: 1 scientist in ergonomics and human factors (EK), and 2 experts on mental health (NW, MBR).

### Search Strategy

Since app stores differ in their acceptance policy and therefore might offer different apps, the study sample was identified through systematic searches in 2 app stores: iTunes and Google Play. The algorithms within Google Play and iTunes work differently in how they classify and rank apps and make matches for specific keywords. For instance, the Google Play algorithm considers the keywords from the description of an app, and it will rank the app in the search results accordingly. The first results listed are the most relevant. In iTunes, the app description does not influence the app store algorithm in ranking the apps.

Between December 2014 and April 2015 apps were searched, screened, and downloaded. Search terms were based on Boolean logic and included combinations for domain (work, worksite, workplace, worker, employee), health (activity, health, lifestyle, stress, mental, physical, behavior, risk, sitting, posture, shiftwork, vitality, resilience, wellbeing), and intervention (coach, intervention, assistant, motivation, support, program). Searches were performed without using the app stores’ categories.

### Inclusion

To be included, apps had to meet the following criteria: (1) be work-related, (2) be aimed at stress prevention or coping and/or psychosocial risk reduction and/or physical risk reduction and/or prevention of sedentary behavior and/or promotion of physical activity, (3) be aimed at healthy adults, (4) provide individually tailored feedback, and (5) be English or Dutch. Apps that contained handbooks, product catalogues or Occupational Safety and Health incident reporting were excluded. Apps that focused on older adults, students or individuals with health problems (eg, depression) were also excluded.

### Screening and Assessment

**Figure 1:**
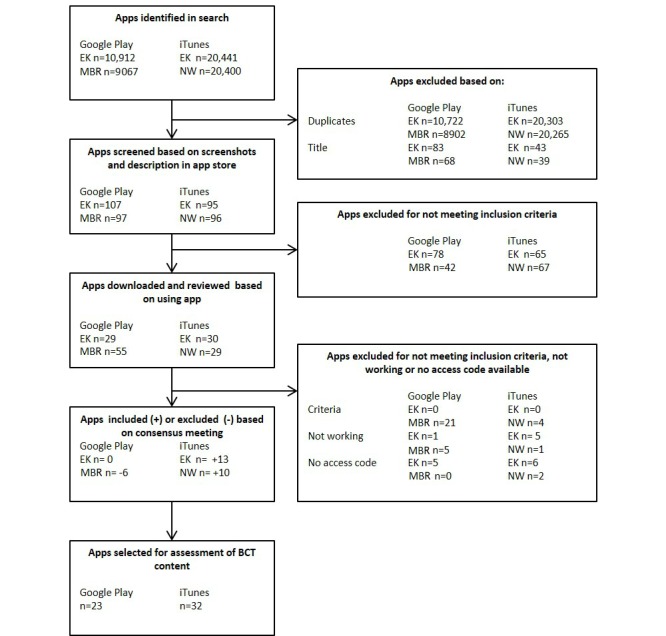
Overview of the selection and screening procedure of apps for assessment of behavior change techniques (BCTs).

Figure 1 shows an overview of the selection and screening procedure.

Search, inclusion, screening of apps, and assessment of BCTs were performed by 3 researchers (EK and NW for iTunes; EK and MBR for Google Play). Any differences were resolved by discussion with the 2 reviewers and if necessary with the third reviewer.

First, search terms were entered in the app stores and apps were searched based on their title. Second, using the screenshots and description in the app store, the apps were screened using the inclusion criteria. Third, if an app seemed to be suitable for inclusion, it was downloaded to an iPhone 4 or a Samsung Galaxy S2. If there were doubts whether an app met the inclusion criteria, it was downloaded. If an app had a free version and a paid version, the free version was downloaded first to be reviewed. If the paid version contained additional features, it was downloaded and used for further analysis. Some apps required a unique access code. In this case, the app providers were contacted by email or phone to request a temporary access code or a demo version. While some app providers cooperated, others did not respond. These apps were not included for further analysis. Fourth, the downloaded apps were again assessed based on the inclusion criteria. Some apps appeared to be not working—these were excluded. In a consensus meeting, the final set of apps for assessment on BCTs was selected.

The reviewers used the included apps until they felt that they were familiar with the details. This varied from one hour (for very basic apps) to four weeks (for extensive apps or apps that took time before the user received feedback). The apps were assessed using the taxonomy of BCTs used in interventions, developed by Abraham and Michie [61]. This taxonomy consists of 26 BCTs and has been previously used to identify BCTs in apps [36,40]. For practical reasons we chose not to use the recent and comprehensive taxonomy by Michie et al with 93 BCTs [62]. This involved a high sensitivity of techniques which were considered too sensitive for the evaluation of apps. The taxonomy of the 26 BCTs formed the basis of the more elaborate taxonomy. In this approach, some of the BCTs used in the earlier taxonomy were specified into more detailed BCTs. To fully understand the content of the 26 BCTs, we studied the 93 BCTs. Before evaluation, all reviewers examined the coding manual and discussed each technique carefully, until a consensus was reached on definitions. Some definitions of BCTs from the taxonomy of Abraham and Michie [61] were adapted to be used for the assessment of apps (Multimedia Appendix 1). For each app, the researchers evaluated and provided a score if the 26 BCTs were present (1) or not (0). In addition to the BCTs, the researchers assessed whether the app was aimed at physical risk prevention, psychosocial risk prevention (including stress prevention or coping) or lifestyle promotion (prevention of sedentary behavior or promotion of physical activity). The apps were scored independently, and Krippendorff alpha was used to evaluate interrater reliability since it can be used regardless of the number of observers, levels of measurement, sample sizes, and the presence or absence of missing data [67]. Also, the app name, a short description, the name of the app store, and the price for each app were collected, and stored in Excel for further analysis. The means and frequencies were calculated for the BCTs and the price of the app. Krippendorff alpha for nominal data was used to evaluate interrater reliability.

## Results

### General Findings

The reviewers detected 10,912 (EK) and 9,067 (MBR) apps in Google Play and 20,441 (EK) and 20,400 (NW) in iTunes. The difference between Google Play and iTunes is because, for each search, Google Play generates a maximum of 250 results per search term, in contrast to iTunes, which has no maximum.

After the inclusion procedure, 45 apps were selected for the assessment of BCTs (see Table 1 for a general overview of the apps). Thirteen apps were found in Google Play, 22 in iTunes, and 10 were found in both app stores. Of the apps found in both stores, iChange2 and Wellmo were evaluated by NW and EK on an iPhone 4. The other 8 found in both stores were evaluated by MBR and EK on a Samsung Galaxy S2. Thirty-two apps were reviewed by NW and EK on an iPhone 4 while MBR and EK reviewed 23 apps on a Samsung Galaxy S2. In total, 45 different apps were evaluated.

Reliability data is shown in Table 1. Krippendorff alpha coefficients ranged from .23 to 1.00. Of the 45 reliability tests, 34 (76%) apps yielded alphas of at least .61 indicating good reliability. Fair reliability was found for 9 (29%) apps, which yielded alphas ranging from .41 to .60. Inferior reliability was assessed for 2 (4%) apps that scored below .41 [68].

Of the 45 apps, 13 (29%) had to be paid for with a mean price of €2.40 (range €0.99-4.99). Twenty-nine apps (64%) were free, and 3 (7%) apps had an access code. This access code was used when the app was offered as part of a company program. These apps are not free; however, the cost of these apps is unknown.

Fifteen (33%) apps were targeted at physical risk prevention, 23 (51%) at psychosocial risk prevention (including stress prevention or coping with stress), and 34 (76%) at lifestyle promotion (prevention of sedentary behavior or promotion of physical activity). Twenty-three (51%) apps were directed at a minimum of two categories, and 22 (49%) at just 1.

### Behavior Change Techniques

The average number of BCTs was 7 (range 2-18). Most BCTs were used in iChange2 (18) and Wellmo (16). Table 1 shows that the least BCTs were identified in Positive Me (2), Ergometer (3), Office health alarm clock (3), and Stress Check by AIIR consulting LLC (3).

**Figure 2:**
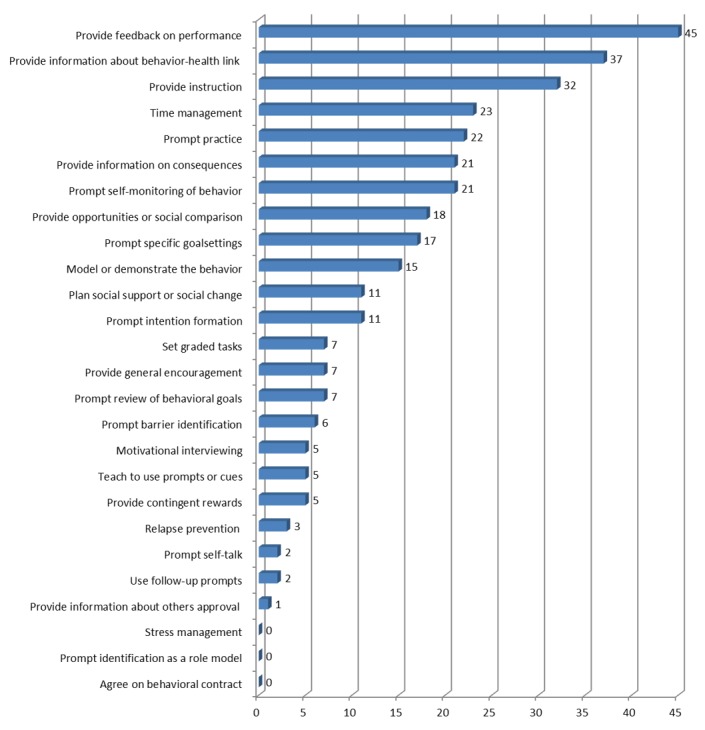
Frequencies of the behavior change techniques found in apps using the taxonomy by Abraham and Michie [61].

**Figure 3:**
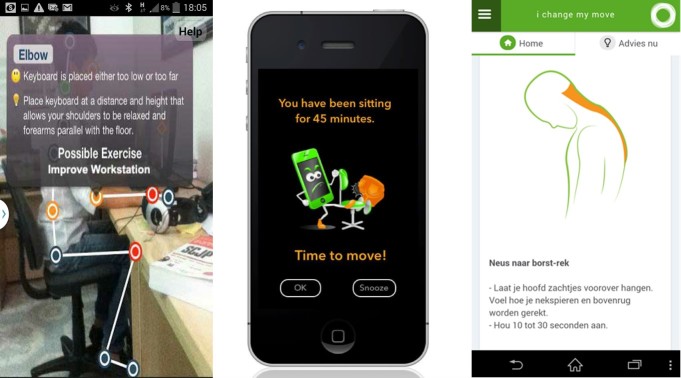
Examples of behavior change techniques used in apps, from left to right: “provide feedback on performance” (Ergo@WSH), “prompt practice” (Get Off Your Butt!) and “model or demonstrate behavior” (iChange2). Pictures have been taken from app descriptions in Google Play store (Ergo@WSH), iTunes (Get Off Your Butt!) and from app provider (iChange2).

Figure 2 shows the BCTs identified most frequently and which BCTs were not. All 45 apps “provided feedback on performance”. This was no surprise since it was one of the inclusion criteria. Other techniques that were used more often were “provide information about behavior-health link” in 37 (82%) apps and “provide instruction” in 32 (71%) apps. Techniques that were used least were “relapse prevention” found in 3 (7%) of the apps, “prompt self-talk” in 2 (4%) apps, “use follow-up prompts” in 2 apps, and “provide information about others approval” in 1 app. “Stress management,” “prompt identification as a role model,” and “agree on behavioral contract” were not used by any of the apps (Figure 3).

Finally, combinations of techniques were analyzed. The combination “provide information about behavior-health link” with “prompt intention formation” was found in 7 (16%) apps (Brightr, iChange2, Move More, Office Buzz, Wellmo, 48-hour stress relief and Office exercise & stretch). The combination “provide information about behavior-health link” with “provide information on consequences” and “use follow-up prompts” was found in 2 (4%) apps (iChange2 and Wellmo). These combinations were found to be the most effective in health behavior change in the meta-analysis by Dusseldorp et al [66] indicating potential effectiveness in mHealth apps. The least effective combination “provide feedback on performance” without “provide instruction,” according to the meta-analysis of Dusseldorp et al [66], was found in 13 (29%) apps (Break Reminder, Darma, Fitlab, iSteplog, My Wellbeing App: Psycare Assist, Office Buzz, Office health alarm clock, Positive Me, Stand-up!, Standing desk companion [Varidesk], Stress Check [AIIR consulting LLC], Walk to Work and Workonit).

**Table 1:** Descriptive data of the apps that were evaluated for the presence of behavior change techniques.

Name of the app	Krippendorff alpha	App store purchased	Price (€) per code	Category of risk prevention or lifestyle promotion that apply to the apps				BCT^a^ score
				Physical risk prevention	Psychosocial risk prevention	Lifestyle promotion		
1-minute desk workout	.59	iTunes	0	Yes	—	Yes	8	
48-hour stress relief	.59	iTunes	4.99	—	Yes	—	8	
Aetna Resources for Living	.83	Google Play / iTunes	0	—	Yes	—	8	
Balance Coach Report Pro	.63	iTunes	2.99	—	Yes	Yes	6	
Break Reminder	1.00	Google Play	0	Yes	Yes	Yes	4	
Brightr	.95	Google Play / iTunes	Access code	—	Yes	Yes	10	
Carecall	.32	iTunes	0	—	Yes	Yes	6	
Chair Yoga	.84	iTunes	2.99	Yes	Yes	Yes	6	
CNV mijn loopbaan app	.79	iTunes	0	—	Yes	Yes	5	
Darma	.76	iTunes	0	—	—	Yes	4	
Desk Workout	.73	iTunes	0	Yes	—	Yes	7	
Ergo@WSH	.77	Google Play / iTunes	0	Yes	—	Yes	7	
ErgoCom	.72	Google Play	0	Yes	—	—	4	
Ergometer	.63	Google Play	0	Yes	—	—	3	
Ergonomics	.86	iTunes	0	Yes	—	Yes	8	
Fatigue Score Calculator	.90	iTunes	1.29	—	—	Yes	5	
Fitlab	.79	Google Play	0	—	Yes	Yes	4	
Get Off Your Butt!	.91	iTunes	1.99	—	—	Yes	6	
Happy@work	1.00	Google Play	3.99	—	Yes	—	5	
Headspace.com meditation	.79	Google Play	0	—	Yes	Yes	11	
Ichange2	.78	Google Play / iTunes	Access code	—	Yes	Yes	18	
iStepLog	.63	iTunes	0	—	—	Yes	11	
Ladies' Office Workout	.55	Google Play / iTunes	0	—	—	Yes	9	
Measure Workplace Stress	.64	Google Play / iTunes	0	—	Yes	—	4	
Minute Stretches	.84	iTunes	0.99	Yes	—	Yes	7	
Move More	.62	iTunes	0.99	—	—	Yes	11	
My Wellbeing App: Psycare Assist	.23	Google Play / iTunes	0	—	Yes	Yes	4	
Office Buzz	.63	Google Play	0	—	Yes	Yes	7	
Office exercise & stretch	.62	Google Play	1.18	—	—	Yes	9	
Office health alarm clock	.43	iTunes	0.99	Yes	—	—	3	
Office Wellness	.92	Google Play	0	Yes	—	Yes	8	
Positive Me	.43	iTunes	0	—	Yes	Yes	2	
Provider resilience	.65	iTunes	0	—	Yes	—	12	
Salute the Desk	.78	iTunes	3.99	Yes	—	Yes	9	
Stand up!	.63	Google Play / iTunes	0	—	—	Yes	8	
Standing desk companion (Varidesk)	.59	iTunes	0	—	—	Yes	7	
Stop Sitting virtual weight loss	.75	iTunes	0.99	—	—	Yes	8	
Stress Check (wisdomathand/office harmony)	.61	Google Play	0	—	Yes	—	4	
Stress Check (AIIR consulting LLC)	.61	iTunes	0	—	Yes	Yes	3	
Stress Releaser Meditation	.61	Google Play	3.82	—	Yes	—	5	
VGZ Mindfulness Coach	.51	Google Play	0	—	Yes	—	8	
Voom	.63	iTunes	0	Yes	—	Yes	11	
Walk to Work	.53	Google Play	0	—	—	Yes	6	
Wellmo	.65	Google Play / iTunes	Access code	Yes	Yes	Yes	16	
Workonit	.50	Google Play / iTunes	0	Yes	Yes	Yes	8	

^a^BCT: behavior change technique.

## Discussion

### Principal Findings

In this study, the presence of BCTs was identified in apps for the mental and physical health of employees. Previously, researchers have studied the presence of BCTs in apps, such as physical activity apps [36,40,54,60,63], dietary apps [53], medication adherence apps [69] or cancer survivorship apps [70]. Others have studied the presence of BCTs in wearable lifestyle activity trackers [71,72]. However, this study was the first to assess BCTs in apps aimed at improving the mental and physical health of employees. Also, this app assessment was the first to look at specific combinations of BCTs in apps, which might serve as an indicator of potential effectiveness.

The majority of the apps (34/45, 76%) in this study aimed to improve the health of employees targeted lifestyle promotion, while the number of apps directed at psychosocial risk prevention (23/45, 51%) and physical risk prevention (15/45, 33%) was much lower. About half (22/45, 49%) of the apps targeted just 1 of these categories. Reviewers noticed that lifestyle apps used sensors more often (eg, the accelerometer of the mobile phone used for step counting). In contrast, apps aiming at psychosocial risk prevention rarely used sensors to monitor; these apps generally used questions or questionnaires to gather data. One of the main advantages of mobile technology compared to the traditional nondigital interventions in the workplace setting is the ability to monitor the user’s behavior with sensors continuously. This offers the opportunity to bring behavioral interventions into an important working context where people make decisions about their health and encounter barriers to behavior change. Differences in technical possibilities might influence the sort of apps that are being developed and the kind of behaviors they target.

The results of this study showed a limited presence of theoretical behavior change constructs. Previous research has highlighted the shortage of the application of behavior change theory in digital interventions, such as websites and apps designed to promote health behavior change [36,40]. Cowan et al [63] suggest that the general lack of theoretical constructs on behavior change included in apps might not be entirely unexpected, given that app developers’ expertise relates to software development and may not include health behavior theory. Therefore, they might not thoroughly incorporate health behavior change theory into their apps [63]. Another explanation for these findings might be that, as per Dusseldorp et al [66], it is the type, quality, and combinations of BCTs, and how they are implemented, rather than the quantity of the techniques that matter. Finally, some techniques might not be detected by the researchers. The low Krippendorff alpha values found in some apps, as well as the discussions, emerged during the consensus meetings showed that reviewers did not always discover all features of the apps. Some features were not explicit during use. For example, reminders, updates, and feedback might have occurred for one reviewer, but not for another. Some BCTs were not easily traceable, for instance only via pop-up messages. This resulted in a different assessment of BCTs and might explain the interrater variability for some of the apps, with the lowest Krippendorff alphas belonging to Carecall and My Wellbeing App: Psycare Assist. However, it is important to note that low Krippendorff alphas might also exist in the case of rare values, especially with binary variables (ie, BCT present or BCT not present) with 1 rare value. Krippendorff alpha compares the “observed” and “expected” disagreements and to satisfy this it takes into account the prevalence of the categories coded for the variable. Nevertheless, one of the strengths of this study is that all apps have been screened and identified by at least two reviewers and in general, reasonable to good interrater reliability has been established.

In apps for mental and physical health, 7 BCTs were identified on average. Also, the number of applied BCTs showed a large variation between apps (range 2-18). These results are in line with those of Middelweerd et al [40] who found an average of 5 (range 2-8), Conroy et al [54] (average 4, range 1-13), Yang et al [60] (average 7, range 1-21), and Direito et al [36] (average 8, range (2-18), although these studies targeted physical activity and nutrition apps).

In this study, it was shown that the most common BCTs in apps for the health promotion of employees were “feedback on performance,” “providing information about the behavior-health link and provide instruction.” Middelweerd et al [40], Direito et al [36], and Conroy et al [54] also showed that “provide feedback on performance” and “provide instruction” were among the most identified BCTs. “Provide feedback on performance” was also found by Middelweerd et al [40] to be the most applied technique, although this was, similar to the current study, one of the inclusion criteria.

The current study showed that BCTs “relapse prevention,” “use follow-up prompts,” “prompt self-talk,” and “provide information about others’ approval” were identified the least. “Relapse prevention” and using “follow-up prompts” are important for sustained behavior change, but in the current study, these were applied in 3 apps only, which might question the value of these apps for changing behavior in the long-term [36]. However, it is unclear why these BCTs have been found in only a limited number in the sample of apps. For instance, these techniques might work well for interventions targeting addictive behaviors (eg, smoking) but might not be relevant for interventions promoting work style or habit formation.

“Stress management,” “prompt identification as a role model,” and “agree on behavioral contract” were not applied at all in any of the apps, which is in line with the work of Middelweerd et al [40] and Direito et al [36]. Further findings were not in line with the work of others: “prompt identification as a role model” was the fourth most applied technique in the study by Direito et al [36] but was not applied in that of Middelweerd et al [40], nor in the current study. “Prompt identification as a role model” was found by Direito et al [36] and there seems to be no technical obstacles to also applying “stress management” and “prompt identification as a role model” in apps. It appears that app developers might lack expertise in health behavior theory and therefore not include these techniques in their apps.

Compared to nondigital interventions in the workplace setting, one of the advantages of apps is the ability to monitor users’ behavior continuously and to deliver context-aware, personalized interventions. Consequently, these technologies support a participative role of users, while enhancing their responsibility for their health and performance [38,41-43]. For this reason, it was expected that many apps in the current study would have applied “prompt self-monitoring” in 21 (47%) apps, “plan social support or change” in 11 (24%) apps, and “prompt barrier identification” in 6 (13%) apps as a technique. The results did not quite confirm these expectations.

Applying certain combinations of BCTs is also essential. Dusseldorp et al [66] concluded from their meta-analysis that specific combinations of BCTs increase the likelihood of achieving change in health behavior, whereas other combinations decrease the possibility. The results of the current study showed that only a few apps applied most effective combinations and many apps applied the least effective. The meta-analyses by Dusseldorp et al [66] were performed with data on nondigital interventions. It is unclear whether this applies to digital interventions as well, but app developers should at least be conscious on how the number, the use, and combinations of BCTs might influence the effectiveness of an app. Therefore, future research should focus on the evaluation of which BCTs and combinations of BCTs are likely to be successful in effectively changing unhealthy behavior. Also, the present study shows that knowledge on effective BCTs might currently be underused in app development and suggests the need for multidisciplinary collaboration between app developers and behavior change experts. Others have concluded this as well [36,63,73]. Besides, to design tailored and targeted app-based interventions, insight into the preferences of the target population for certain BCTs is of importance. This has been shown by Belmon et al [74] for young adults in physical activity apps. Some BCTs were rated as more positive to apply than others. Ratings of BCTs differed according to personality traits and exercising self-efficacy. This may apply to apps for employees, and therefore, preferably employees should also be engaged in the development.

This study on BCTs in apps for the mental and physical health of employees had certain limitations. The procedure to search, identify, and review apps is susceptible to bias. Reviewers searched, screened, and downloaded apps on different days. Generally, apps are developed very fast and what is offered in app stores varies daily. This might have influenced the search results, especially those based on algorithm ranking (Google Play).

These fast developments also became apparent when some apps that were selected for download appeared to be untraceable. Presumably, many new apps have also appeared in the meantime. Still available apps have likely been changed, and new versions are available in the app stores since apps are updated continuously. This is illustrated by the study of Larsen et al [75] on the availability of mental health apps in iTunes and Google Play stores. They found 50% of search results changing within 4 months and an app being removed every 2.9 days. Therefore, conclusions on the apps that participated in the current study have to be interpreted with caution.

The taxonomy of Abraham and Michie [61] has not been developed specifically for apps. Therefore, reviewers had to translate the BCTs to app characteristics, which might have led to different interpretations than initially intended. For instance, stress management appeared to be a difficult BCT to interpret. It is defined as “may involve a variety of specific techniques (eg, progressive relaxation) that do not target the behavior but seek to reduce anxiety and stress.” However, in many apps in this study, management of stress was the targeted behavior, which was confusing. After a consensus meeting, it was decided to identify this technique only in cases where advice was given on ways to facilitate performance of the targeted behavior.

In addition to methodological limitations, there are also limitations in interpreting the results. As stated in the introduction, the extent to which apps are built upon theoretical models of the themes they address is essential (ie, stress management apps making use of evidence-based stress models). The current study focused on the presence of specific combinations of behavior change theories in apps. However, this is not necessarily an indication of good quality. Some of the apps in the study applied BCTs but also gave feedback that was not in line with current scientific insights. This raises the question of the value of these apps in supporting the user to enhance mental and physical health. Although an app might use principles and constructs underpinning the processes of behavior change, it also needs to be consistent with evidence-based practices. Therefore, designing useful apps requires the application of expertise from diverse fields and would benefit from interdisciplinary collaboration. While there is a consensus among software developers on the importance of engaging users, an mHealth app for employees would also benefit from collaboration with behavior change experts and experts in mental and physical health [76].

Moreover, the current study does not answer the question of whether apps are effective in changing behavior and thereby in the prevention of physical and mental health risk or promotion of a healthy lifestyle. To determine effectiveness, controlled trials are necessary, preferably using evaluation methods that fit with the fast, iterative development processes of apps (eg, a stepped wedge design) [35,37]. To date, the evidence base of apps is still scarce. Many apps are not based on solid evidence or evaluated with scientific methods [54,63,73].

Despite these limitations, this study provides the first analysis of health behavior theory applied in apps for the mental and physical health of employees. This research method cannot establish effectiveness and usability of these apps. Further research is needed to assess the effectiveness and usability of apps as intervention means for employees.

### Conclusion

The findings of this study suggest that apps might be substantially improved to bring behavioral interventions into the working context where employees make decisions about their health and encounter barriers to behavior change. This study might be a first step toward implementing BCTs in a manner that is likely to increase behavior change potential.

The results, in general, showed a limited presence of BCTs, limited use of potentially successful combinations of BCTs in apps, and the use of potentially unsuccessful combinations of BCTs. Current knowledge on potentially effective combinations of BCTs seems to be underused in app development for the occupational setting. Knowledge of BCTs should be incorporated more in the development of apps. Combining behavior change theory and providing content with a robust evidence base and taking into account the specific context of the occupational setting could contribute to the development of effective mHealth-based interventions for employees and decrease the burden of work-related diseases. Although BCTs have been shown to be effective in face-to-face or online behavior change interventions, it is still unclear whether they are effective mHealth interventions. Future research should, therefore, focus on evaluating which BCTs and combinations of BCTs are effective in changing health behavior of employees when used in apps. For this evaluation, quantitative and qualitative methods should be used.

To increase potential and effectiveness, a collaboration between app developers, health behavior change professionals, experts on physical and mental health, and end-users is suggested. Combinations of expertise could provide higher quality apps. Until now, it is unclear which criteria could be used by organizations when selecting apps to offer to their employees. Furthermore, for employees, it remains unclear which app would help them best to improve their physical and mental health at work. An increase in knowledge on the effectiveness of BCTs in apps could be used to develop guidelines for app developers and the development of selection criteria for companies and individuals.

## Appendix

Multimedia Appendix 1 Definitions of behavior change techniques.

## Abbreviations

BCT: behavior change technique
mHealth: mobile health
