# Supplementary material for: Behavior Change Techniques in mHealth Apps for the Mental and Physical Health of Employees: Systematic Assessment
Source: JMIR Mhealth Uhealth. 2018 Oct 3;6(10):e167. doi: 10.2196/mhealth.6363 (PMC6231882; doi:10.2196/mhealth.6363)
Supplement: Multimedia Appendix 1 [file mhealth_v6i10e167_app1.pdf]

Multimedia Appendix 1. Definitions of Behavior Change Techniques

This is a Multimedia Appendix to a full manuscript published in the JMIR mHealth and uHealth. For full copyright and citation information see http://mhealth.jmir.org
doi:10.2196/mhealth.6363

Below, definitions of Behavior Change Techniques, used to evaluate the apps in the current study. Definitions are based on the work of Abraham & Michie (2008) and adapted to be used for evaluation apps. Adaptions are shown in *italics*

**Definitions of Behavior Change Techniques (BCTs)**	
1. Provide information about behavior health link	General information about behavioral risk, for example, susceptibility to poor health outcomes or mortality risk in relation to the behavior.
2. Provide information on consequences	Information about the benefits and costs of action or inaction, focusing on what will happen if the person does or does not perform the behavior.
3. Provide information about others' approval	Information about what others think about the person's behavior and whether others will approve or disapprove of any proposed behavior change.
4. Prompt intention formation	Encouraging the person to decide to act or set a general goal, for example, to make a behavioral resolution such as "I will take more exercise next week".
5. Prompt barrier identification	Identify barriers to performing the behavior and plan ways of overcoming them.
6. Provide general encouragement	Praising or rewarding the person for effort or performance without this being contingent on specified behaviors or standards of performance.
7. Set graded tasks	Set easy tasks, and increase difficulty until target behavior is performed.
8. Provide instruction	Telling the person how to perform a behavior and/or preparatory behaviors.
9. Model/ demonstrate the behavior	An expert shows the person how to correctly perform a behavior, for example, in class or on video, *or by visualizations in the app*.
10. Prompt specific goal setting	Involves detailed planning of what the person will do, including a definition of the behavior specifying frequency, intensity, or duration and specification of at least one context, that is, where, when, how, or with whom.
11. Prompt review of behavioral goals	Review and/or reconsideration of previously set goals or intentions
12. Prompt self-monitoring of behavior	The person is asked to keep a record of specified behavior(s) (e.g., in a diary). *An app that requires pressing a button at every intake would also count as an instance of self report. Multiple time-points.*
13. Provide feedback on performance	Providing data about recorded behavior or evaluating performance in relation to a set standard or others' performance, i.e., the person received feedback on their behavior. *Includes providing an overview of recorded behavior*
14. Provide contingent rewards	Praise, encouragement, or material rewards that are explicitly linked to the achievement of specified behaviors.

15. Teach to use prompts/ cues	Teach the person to identify environmental cues that can be used to remind them to perform a behavior, including times of day or elements of contexts.
16. Agree behavioral contract	Agreement (e.g., signing) of a contract specifying behavior to be performed so that there is a written record of the person's resolution witnessed by another.
17. Prompt practice	Prompt the person to rehearse and repeat the behavior or preparatory behaviors.
18. Use follow up prompts	Contacting the person again after the main part of the intervention is complete.
19. Provide opportunities for social comparison	Facilitate observation of non-expert others' performance for example, in a group class or using video or case study *or via app.*
20. Plan social support/ social change	Prompting consideration of how others could change their behavior to offer the person help or (instrumental) social support, including 'buddy' systems and/or providing social support.
21. Prompt identification as role model	Indicating how the person may be an example to others and influence their behavior or provide an opportunity for the person to set a good example.
22. Prompt self-talk	Encourage use of self-instruction and self-encouragement (aloud or silently) to support action.
23. Relapse prevention	Following initial change, help identify situations likely to result in readopting risk behaviors or failure to maintain new behaviors and help the person plan to avoid or manage these situations.
24. Stress management	May involve a variety of specific techniques (e.g. progressive relaxation) that do not target the behavior but seek to reduce anxiety and stress.
25. Motivational interviewing	Prompting the person to provide self-motivating statements and evaluations of their own behavior to minimize resistance to change.
26. Time management	Helping the person make time for the behavior (e.g., to fit it into a daily schedule).

Reference

Abraham C & Michie S. (2008). A taxonomy of behavior change techniques used in interventions. Health Psychology; 27(3):379–87
